# A Widening Gap? Changes in Multiple Lifestyle Risk Behaviours by Socioeconomic Status in New South Wales, Australia, 2002–2012

**DOI:** 10.1371/journal.pone.0135338

**Published:** 2015-08-20

**Authors:** Ding Ding, Anna Do, Heather-Marie Schmidt, Adrian E. Bauman

**Affiliations:** 1 Prevention Research Collaboration, Sydney School of Public Health, The University of Sydney, Camperdown, New South Wales, Australia; 2 Centre for Epidemiology and Evidence, New South Wales Ministry of Health, North Sydney, New South Wales, Australia; 3 Centre for Population Health, New South Wales Ministry of Health, North Sydney, New South Wales, Australia; TNO, NETHERLANDS

## Abstract

**Background:**

Socioeconomic inequalities in health outcomes have increased over the past few decades in some countries. However, the trends in inequalities related to multiple health risk behaviours have been infrequently reported. In this study, we examined the trends in individual health risk behaviours and a summary lifestyle risk index in New South Wales, Australia, and whether the absolute and relative inequalities in risk behaviours by socioeconomic positions have changed over time.

**Methods:**

Using data from the annual New South Wales Adult Population Health Survey during the period of 2002–2012, we examined four individual risk behaviours (smoking, higher than recommended alcohol consumption, insufficient fruit and vegetable intake, and insufficient physical activity) and a combined lifestyle risk indicator. Socioeconomic inequalities were assessed based on educational attainment and postal area-level index of relative socio-economic disadvantage (IRSD), and were presented as prevalence difference for absolute inequalities and prevalence ratio for relative inequalities. Trend tests and survey logistic regression models examined whether the degree of absolute and relative inequalities between the most and least disadvantaged subgroups have changed over time.

**Results:**

The prevalence of all individual risk behaviours and the summary lifestyle risk indicator declined from 2002 to 2012. Particularly, the prevalence of physical inactivity and smoking decreased from 52.6% and 22% in 2002 to 43.8% and 17.1% in 2012 (*p* for trend<0.001). However, a significant trend was observed for increasing absolute and relative inequalities in smoking, insufficient fruit and vegetable consumption, and the summary lifestyle risk indicator.

**Conclusions:**

The overall improvement in health behaviours in New South Wales, Australia, co-occurred with a widening socioeconomic gap.

**Implications:**

Governments should address health inequalities through risk factor surveillance and combined strategies of population-wide and targeted interventions.

## Introduction

Lifestyle risk behaviours, such as smoking, diet, and physical inactivity, are important contributors to morbidity and mortality worldwide [1]. Studies have found that the co-occurrence of multiple health risk behaviours could exert synergistic effects on health [2]. Furthermore, these risk behaviours consistently cluster among certain population subgroups, such as those with lower socioeconomic status (SES) [3, 4]. The interconnected nature of health risk behaviours has led to some governments adopting an integrated approach to monitoring lifestyle risk factors [4, 5], including a “Dashboard of Indicators” for chronic disease prevention proposed in 2003 by New South Wales (NSW) Ministry of Health in Australia [4].

In the last few decades, public health interventions in many countries around the world, including in Australia, have led to population-level improvement in some individual health behaviours. However, there is a concern that population-level interventions may widen socioeconomic inequalities, at least initially, because advantaged subgroups have better access to resources and are more likely to adopt healthful behavioural change [6]. Furthermore, evidence suggests that relative socioeconomic inequalities in health outcomes have increased over the last few decades in some developed countries including in the USA [7], Europe [8], and elsewhere [9]. Meanwhile, studies have found that combinations of behavioural risk factors explain a large proportion of the socioeconomic differences in health [10, 11]. To date, a small number of studies have examined how socioeconomic inequalities in individual health risk behaviours evolved overtime and found widening gaps between those of high and low SES, a pattern echoing that of health outcomes [5, 12]. However, the issue about health inequalities is highly context-specific and analyses from other countries are needed to inform relevant health initiatives and to gain global perspectives on socioeconomic inequalities in health.

Australia has one of the highest life expectancies in the world [13], however, similar to many other countries, health outcomes of Australians differ noticeably across socioeconomic subgroups, and there is evidence for widening relative health inequalities despite improving absolute SES gaps in health [14]. The only Australian study to our knowledge that examined temporal trends in inequalities of health risk behaviours found that the relative inequalities in smoking and physical inactivity had increased between 1989–1990 and 2001 [15]. However, analyses of trends beyond 2001 and of a broader range of lifestyle risk behaviours are urgently needed in Australia. Herein we used population representative data from NSW, Australia to examine 1) the trends in individual health risk behaviours and combined lifestyle risk from 2002 to 2012, and 2) whether the inequalities in risk behaviours by socioeconomic positions have changed over this time period.

## Methods

### Data source

Data were sourced from the NSW annual Adult Population Health Survey during the period of 2002 to 2012, a computer assisted telephone interview survey that collects information on the health behaviours and outcomes of adults [16]. The NSW Adult Population Health Survey has been administered by the NSW Ministry of Health annually since 2002. NSW residents aged 16 years and over were randomly sampled from each health administrative area using list-assisted random digit dialling. The survey is approved by the NSW Population and Health Services Research Ethics Committee. Participants provided verbal informed consent at the start of the interview and the information related to the participants were de-identified and anonymised. Since 2012, the survey started to include mobile phone-only users in addition to the sampling of landline phone users [17]. Over time, the response rates (calculated as the total number of complete interviews divided by the sum of complete interviews, partial interviews, refusals, non-contact, and other) of the survey stayed stable around 45% and the cooperation rates (calculated as total number of complete interviews divided by the sum of complete interview, partial interview, and refusal) have remained around 65% [18].

### Measures

Questionnaires for data collection are available on the survey website [19]. Socio-demographic characteristics and lifestyle risk behaviours were reported by the participants. Socioeconomic status was operationalised using an individual-level measure of self-reported educational attainment, which was categorised as ≤school certificate (10 years), higher school/trade/diploma (12 years), and ≥university, and area-level socioeconomic disadvantage, measured by the Index of Relative Socio-economic Disadvantage (IRSD). The IRSD is an aggregate measure that summarises information about the economic and social conditions of people and households at the postal area level, including educational attainment, employment, occupation, income, English language proficiency, dwellings, car ownership, and several other indicators. Scores were ranked within NSW and categorised into quintiles from the least to the most disadvantaged postal areas [20].

Four lifestyle risk behaviours were included in the current analysis. Smoking risk was defined as being a current smoker (this included self-report of daily and occasional smoking). Alcohol risk was defined as consuming more than 14 standard drinks per week (one standard drink is defined as a drink containing 10 grams of alcohol, which approximates one glass of wine, one half pint of beer, or one shot of spirits); this is consistent with exceeding the Australian recommendations for alcohol consumption [21]. Physical activity was measured using the Active Australia Survey, which was previously found to have acceptable reliability and validity [22]. Those who did not meet the minimal recommendation of 150 minutes of physical activity per week over five separate occasions (with vigorous physical activity weighted by two) were defined as at-risk [23]. Dietary risk was measured as consuming less than two servings of fruit and/or less than three servings of vegetables a day. Although the Australian Dietary Guidelines recommend five or more servings of vegetables [24], the very low percentage of the population meeting this recommendation prompted the NSW Department of Health to use three servings of vegetable to monitor consumption trends [25]. Typical for this field of research [2], a summary lifestyle risk index was created as the total number of risk behaviours. This index served as an indicator for the overall lifestyle risk. Based on a previous Australian study, we defined having two risk behaviours or more as having ‘high lifestyle risk’ [26].

### Statistical analysis

Prevalence of individual risk behaviours and having high overall lifestyle risk was estimated for each year using the SAS survey procedure, which weighted each year’s data to be population representative. In 2007, a different split questionnaire design was trialled [27], whereby participants were randomly allocated different modules of questions because the survey was too long to ensure completion rates. This resulted in very few participants (5.5%) answering all four lifestyle questions, consequently, data from 2007 were excluded from the main analyses. A similar split questionnaire design was used in 2008, but a larger percentage of participants were asked multiple lifestyle questions (41% answered all four questions). Because those who were allocated these lifestyle questions were considered a random subsample of the 2008 participants, we retained data from 2008 in our analysis.

To test trends for individual risk behaviours and for having overall high lifestyle risk, models with year as a continuous and a categorical variable were tested and a likelihood ratio test was performed to determine the linearity of the trend. Inequalities were measured using a simple “range” approach by contrasting the most and least disadvantaged subgroups, as discussed by Mackenbach and Kunst [28], based on both educational attainment (≤school certificate vs. ≥university) and the IRSD (Quintile 5 vs. Quintile 1). The sample was weighted to adjust for differences in the probability of selection and to match the age and sex structure of the NSW population. Estimates were standardised to the sex and age structure of the NSW population in 2012 (the most recent year of data) to account for demographic changes within the population. Standard errors were adjusted for the stratified sampling design. Prevalence difference (PD, a measure of absolute inequalities) and prevalence ratio (PR, a measure of relative inequalities) with 95% confidence limits were generated to quantify the differences between the most and least disadvantaged socioeconomic subgroups. Both indicators were used because absolute and relative measures reflect different aspects of inequalities and both should be monitored [28, 29].

To test whether the magnitude of absolute inequalities between the two ends of the SES continuum changed over time, a trend test was performed. To test whether the relative inequalities have changed over time, a multiple logistic regression for survey data analysis was used. The models included a dichotomous outcome (having an individual risk behaviour or scoring 2 or higher on the lifestyle risk index) and independent variables: year, socioeconomic status (education categories or the IRSD quintiles), and an interaction term between year and socioeconomic status. Linearity of the trend was tested by entering year as both a continuous and a categorical variable and performing a likelihood ratio test to compare the two models. The interaction term represents the effect of time on lifestyle risk behaviour for the most disadvantaged subgroup relative to the least disadvantaged subgroup. The sample was weighted and standardised, as previously described.

## Results

### Sample characteristics

From 2002 to 2012, a total of 125,561 participants were interviewed, ranging from 7,962 to 13,205 per year (Table 1). The mean age across survey years ranged from 51 years (SD = 18 years) to 57 years (SD = 17 years). Male respondents made up 37 to 42% of the survey samples. The proportion of participants with a school certificate education declined from 38% in 2002 to 30% in 2012, and the proportion of those reporting a university degree increased from 19% to 29%. Approximately 8–15% of the samples belonged to the least disadvantaged quintile of the IRSD within NSW and 19–27% belonged to the most disadvantaged quintile.

**Table 1:** Socio-demographic and lifestyle characteristics of the NSW Population Health Survey sample (aged 16 years and over) by year, 2002–2012.

Characteristics	Year^a^									
	2002	2003	2004	2005	2006	2008	2009	2010	2011	2012
Age (mean; SD)	51 (18)	51 (18)	52 (18)	53 (18)	53 (18)	55 (18)	55 (18)	56 (17)	57 (17)	54 (18)
Males (%)	42.1	41.1	41.0	40.0	40.7	40.0	38.2	37.7	37.0	41.1
Educational attainment (%)										
≤school certificate	38.1	37.3	33.5	31.2	28.9	37.2	36.1	35.5	35.6	30.4
higher school/trade/diploid	41.5	42.3	44.9	44.7	45.6	37.8	37.2	37.6	37.1	39.4
≥ university degree	19.4	19.6	20.1	22.5	23.6	23.7	25.4	25.8	26.2	29.0
Missing^b^	1.0	0.9	1.5	1.7	1.9	1.3	1.3	1.2	1.1	1.1
Index of relative socio-economic disadvantage (IRSD) (%)										
Quintile 1 (least disadvantaged)	7.6	8.0	11.2	13.8	14.6	14.9	15.2	14.8	14.5	13.8
Quintile 2	13.0	12.8	20.9	17.0	18.7	18.0	18.1	18.5	19.3	18.8
Quintile 3	21.1	20.5	20.1	22.8	22.3	23.6	22.7	22.4	21.8	21.6
Quintile 4	30.9	36.4	25.4	23.3	23.2	24.4	24.3	24.9	22.3	24.1
Quintile 5 (most disadvantaged)	27.5	22.4	21.8	18.5	19.6	18.9	19.5	19.0	21.7	21.3
Missing	0.0	0.0	0.5	4.6	1.6	0.2	0.3	0.4	0.4	0.4
Higher than recommended alcohol consumption^c^ (%)										
No	90.2	89.8	90.1	91.0	90.1	74.8	89.8	91.2	91.2	91.6
Yes	9.1	9.8	9.2	8.2	9.2	7.4	9.5	7.9	8.2	7.6
Missing	0.7	0.4	0.8	0.8	0.7	17.8	0.8	0.9	0.7	0.8
Insufficient physical activity^d^ (%)										
No	44.5	41.9	48.9	48.2	48.6	39.5	48.1	46.4	47.3	48.2
Yes	55.5	58.1	51.1	51.0	46.6	38.3	45.0	46.0	47.8	45.8
Missing	0.0	0.0	0.0	0.9	4.9	22.2	6.9	7.6	4.9	6.0
Insufficient fruit and vegetable intake^e^ (%)										
No	25.4	27.1	24.1	28.9	30.9	27.1	33.6	32.3	29.3	28.2
Yes	72.9	71.5	74.4	69.9	67.0	54.1	63.4	63.9	67.8	67.8
Missing	1.6	1.4	1.5	1.2	2.1	18.8	2.9	3.9	2.8	4.1
Current smoking (%)										
No	79.6	79.3	79.3	81.6	83.4	71.4	84.8	85.3	85.9	84.8
Yes	20.4	20.7	20.6	18.4	16.6	13.7	15.0	14.6	13.9	15.0
Missing	0.0	0.1	0.1	0.1	0.1	15.0	0.2	0.1	0.1	0.1
Summary lifestyle risk index score (%)										
Low/moderate (0–1)	45.0	44.4	46.6	49.7	50.0	22.8	50.6	49.4	49.5	49.4
High (≥2)	52.8	53.9	51.2	47.5	42.8	18.1	39.7	39.8	42.9	41.2
Missing	2.2	1.8	2.3	2.8	7.2	59.1	9.6	10.8	7.6	9.5
Total respondents (n)	12,621	13,008	9,786	11,500	7,962	10,296	10,719	10,245	13,041	13,205

*Source*: *NSW Population Health Survey (unweighted)*, *Centre for Epidemiology and Evidence*, *NSW Ministry of Health*.

^a^ Estimates for 2007 are not reported because the survey split questionnaire design used in this year was not consistent with other years.

^b^ Includes observations with responses “Don’t know”, “Refused” and “Not asked”.

^c^ Defined as more than 14 alcoholic drinks per week.

^d^ Insufficient physical activity for health was defined as less than 150 minutes of moderate-to-vigorous physical activity and/or less than 5 sessions of moderate-to-vigorous physical activity per week.

^e^ Defined as less than 2 servings of fruit and/or less than 3 serves of vegetables per day.

### Trends in individual health risk behaviour and high lifestyle risk

The prevalence of all individual risk behaviours and a score of 2+ on the summary lifestyle risk index declined in a linear fashion from 2002 to 2012 (Table 2). The differences between 2002 and 2012 were particularly noticeable for insufficient physical activity, where the prevalence decreased from 52.6% (95% CI: 51.5–53.8%) to 43.8% (95% CI: 41.9–45.8%), and for current smoking, which declined from 22% (95% CI: 21–23%) to 17.1% (95% CI: 15.6–18.6%). In addition, the prevalence of high overall lifestyle risk (scoring 2 or higher on the lifestyle risk index) decreased by about 10 percentage points, from 54.7% (95% CI: 53.5–55.9%) in 2002 to 45.2% (95% CI: 43.2–47.2%) in 2012 (p<0.001).

**Table 2:** Prevalence^a^ (% with 95% confidence limits) of individual and summary health risk behaviours by year, persons 16 years and over, 2002–2012, NSW, Australia.

Year^b^	High alcohol consumption^c^	Insufficient physical activity^d^	Insufficient fruit and vegetable intake^e^	Current smoking	High lifestyle risk^f^ (≥ 2 risk behaviours)
2002	9.4 (8.7–10.1)	52.6 (51.5–53.8)	77.0 (76.1–78.0)	22.0 (21.0–23.0)	54.7 (53.5–55.9)
2003	10.4 (9.7–11.1)	54.7 (53.6–55.9)	75.4 (74.4–76.3)	22.7 (21.7–23.7)	55.4 (54.2–56.5)
2004	10.0 (9.1–10.8)	47.4 (46.0–48.7)	78.0 (76.9–79.1)	22.3 (21.1–23.4)	52.3 (51.0–53.7)
2005	8.7 (8.0–9.3)	46.8 (45.6–48.0)	75.2 (74.2–76.2)	20.8 (19.8–21.8)	49.3 (48.1–50.5)
2006	10.0 (9.1–10.8)	44.8 (43.4–46.2)	72.9 (71.7–74.1)	18.3 (17.2–19.4)	46.7 (45.2–48.1)
2008	9.8 (9.0–10.7)	44.5 (43.1–46.0)	71.7 (70.5–73.0)	19.1 (18.0–20.3)	45.5 (43.5–47.4)
2009	10.5 (9.7–11.3)	43.8 (42.5–45.1)	70.5 (69.4–71.7)	17.8 (16.8–18.8)	45.4 (44.1–46.8)
2010	8.3 (7.6–9.1)	44.3 (42.8–45.7)	72.0 (70.8–73.2)	16.8 (15.7–17.9)	45.0 (43.5–46.4)
2011	8.5 (7.6–9.4)	45.4 (44.0–46.9)	73.2 (72.0–74.5)	14.7 (13.6–15.8)	45.7 (44.3–47.2)
2012	7.7 (6.7–8.6)	43.8 (41.9–45.8)	73.8 (72.1–75.5)	17.1 (15.6–18.6)	45.2 (43.2–47.2)

*Source*: *NSW Population Health Survey*, *Centre for Epidemiology and Evidence*, *NSW Ministry of Health*.

^a^ Sample adjusted for age and sex structure of NSW population and probability of selection for inclusion in survey; estimates standardised to age and sex structure of NSW population for 2012; estimates exclude persons with responses “Don’t know”, “Refused” or “Not asked”.

^b^ Estimates for 2007 are not reported because the survey split questionnaire design used in this year was not consistent with other years.

^c^ Defined as more than 14 alcoholic drinks per week.

^d^ Defined as less than 150 minutes of moderate-to-vigorous physical activity and/or less than 5 sessions of moderate-to-vigorous physical activity per week.

^e^ Defined as less than 2 servings of fruit and/or less than 3 serves of vegetables per day.

^f^ Defined as having two or more of the preceding lifestyle risk behaviours.

### Trends in inequalities

#### Individual risk behaviours

**Fig 1:**
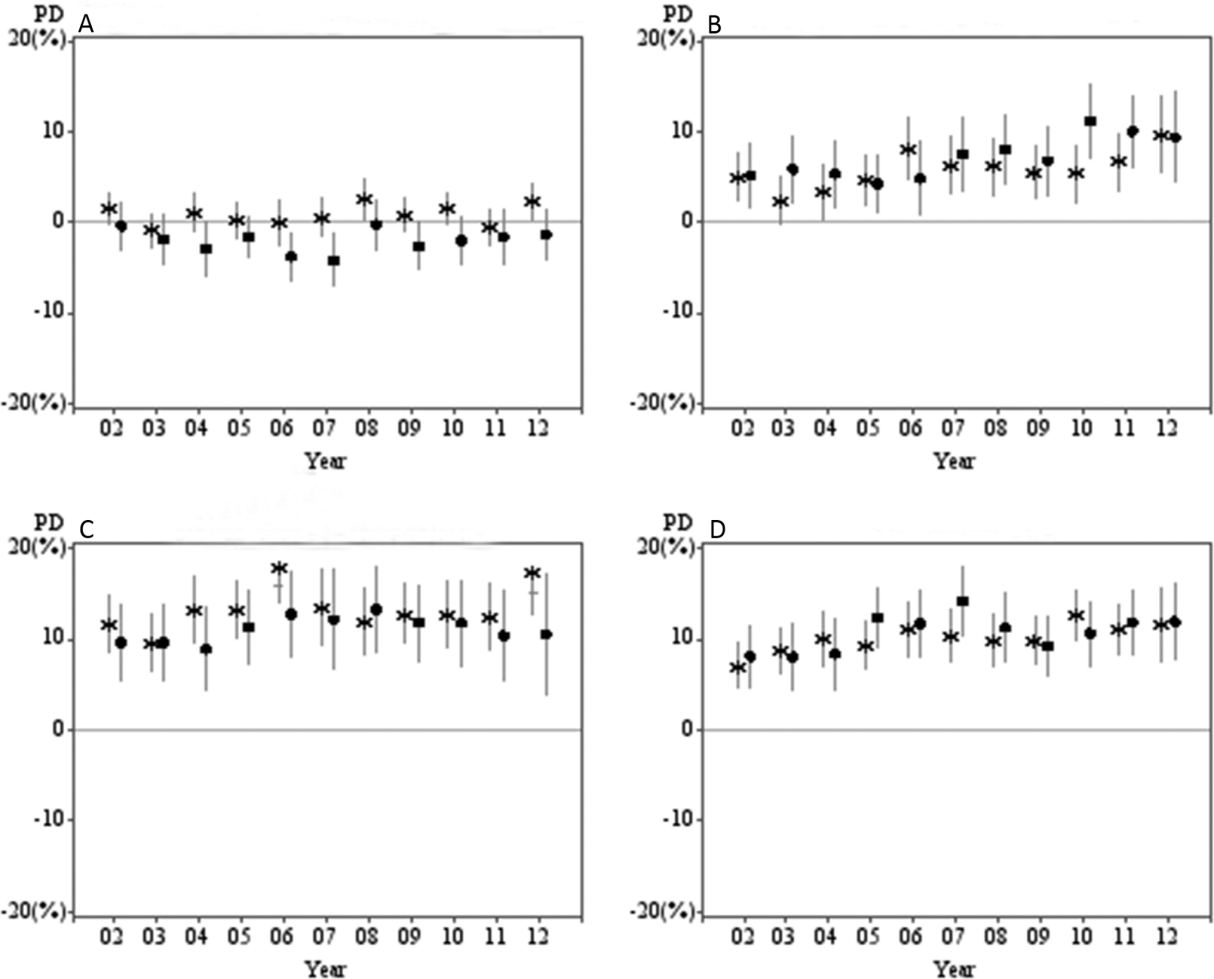
Prevalence difference (PD) of individual health behaviours by educational attainment and index of relative socio-economic disadvantage (IRSD). (A) Higher than recommended alcohol consumption. (B) Insufficient fruit and vegetable intake. (C) Insufficient physical activity. (D) Current smoking. (*by educational attainment • by ISRD)

**Fig 2:**
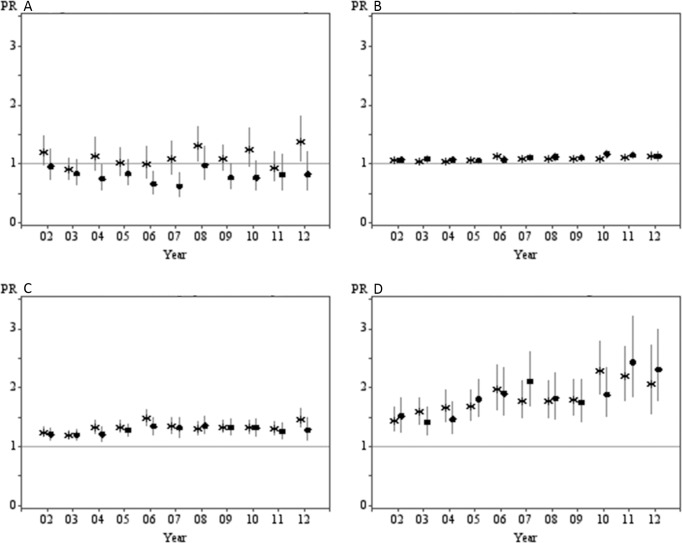
Prevalence ratios (PR) of individual health behaviours by educational attainment and index of relative socio-economic disadvantage (IRSD). (A) Higher than recommended alcohol consumption. (B) Insufficient fruit and vegetable intake. (C) Insufficient physical activity. (D) Current smoking. (*by educational attainment • by ISRD)

Fig 1 presents the prevalence difference (PD) and Fig 2 presents the prevalence ratio (PR) of each individual risk behaviour by socioeconomic subgroup from 2002 to 2012. For alcohol risk, there was a lack of absolute or relative inequalities between the most and least disadvantaged socioeconomic subgroups by education or IRSD, as indicated by a lack of consistent difference between subgroups. For physical inactivity, there were persistent absolute and relative inequalities between 2002 and 2012 but there was no clear indication of an increasing or decreasing trend over time. Meanwhile, a significant trend was observed for increased absolute inequalities in smoking rates, where the PD between the least and most advantageous educational categories had increased from 7% in 2002 to 12% in 2012 (*p for trend*<0.05). A similar increasing trend was observed for relative inequalities based on both education (*p for interaction* = 0.001) and the IRSD (*p for interaction* <0.001). Furthermore, absolute inequalities between education and IRSD subgroups for insufficient fruit and vegetable intake increased over time (*p for trend*<0.05). This increasing trend was also echoed by measures of relative inequalities based on both educational attainment (*p for interaction* = 0.047) and IRSD (*p for interaction* = 0.005).

#### Overall lifestyle risk

High overall lifestyle risk (defined as having two or more lifestyle risk behaviours) was associated with both educational attainment and the IRSD (Table 3). The magnitude of this association increased consistently from 2002 to 2012, as indicated by both PD and PR. For education, the PR for the most disadvantaged subgroup, relative to the least disadvantaged subgroup, was 1.24 (95% CI: 1.16–1.32) in 2002 (PD = 11.1%) and 1.53 (95% CI: 1.37–1.70) in 2012 (PD = 18.5%). Similarly, inequalities between subgroups were also observed with the IRSD measure, where the PR was 1.20 (95% CI: 1.10–1.30) in 2002 (PD = 9.8%) and 1.51 (95% CI: 1.30–1.75) in 2012 (PD = 18.0%). Trend analyses indicated increasing absolute inequalities by education (*p for trend* = 0.035) and IRSD over time (*p for trend*<0.01), and results from survey logistic regression suggested that there was a linear trend of increasing relative inequalities over time by education (*p for interaction* = 0.004) and by IRSD (*p* for interaction = 0.010).

**Table 3:** Prevalence, prevalence ratios, and prevalence differences of high lifestyle risk^a^ for the most and least disadvantaged groups for education and index of relative socio-economic disadvantage (IRSD), by year, persons 16 years and over, 2002–2012, NSW.

Year^b^	Prevalence of having two or more lifestyle risk behaviours (%)^c^		Prevalence ratio^d^	Prevalence difference
	Most disadvantaged^e^	Least disadvantaged^f^	Most/least disadvantaged	Most–least disadvantaged
*Educational attainment*				
2002	57.2 (55.3–59.1)	46.1 (43.6–48.6)	1.24 (1.16–1.32)	11.1 (8.0–14.2)
2003	57.8 (55.9–59.7)	50.0 (47.6–52.5)	1.16 (1.09–1.23)	7.8 (4.7–10.9)
2004	56.9 (54.6–59.2)	43.4 (40.5–46.2)	1.31 (1.21–1.42)	13.6 (9.9–17.2)
2005	53.8 (51.7–56.0)	40.4 (38.1–42.8)	1.33 (1.24–1.43)	13.4 (10.2–16.6)
2006	53.3 (50.6–56.0)	37.1 (34.4–39.9)	1.44 (1.31–1.57)	16.2 (12.3–20.0)
2008	50.2 (46.9–53.4)	39.9 (36.2–43.7)	1.26 (1.12–1.41)	10.2 (5.3–15.2)
2009	51.6 (49.4–53.8)	37.5 (35.0–40.0)	1.37 (1.27–1.49)	14.1 (10.8–17.4)
2010	51.5 (49.0–53.9)	37.6 (34.8–40.4)	1.37 (1.25–1.49)	13.8 (10.1–17.5)
2011	52.5 (50.0–55.0)	37.8 (35.2–40.5)	1.39 (1.27–1.51)	14.7 (11.0–18.3)
2012	53.9 (49.8–57.9)	35.3 (32.6–38.1)	1.53 (1.37–1.70)	18.5 (13.7–23.4)
*Index of relative socio-economic disadvantage (IRSD)*				
2002	58.6 (56.3–60.9)	48.8 (45.2–52.5)	1.20 (1.10–1.30)	9.8 (5.5–14.1)
2003	59.9 (57.4–62.4)	50.2 (46.8–53.6)	1.19 (1.10–1.29)	9.7 (5.4–13.9)
2004	58.5 (55.5–61.5)	47.6 (44.1–51.1)	1.23 (1.12–1.34)	10.9 (6.3–15.5)
2005	55.9 (53.1–58.6)	43.7 (40.8–46.6)	1.28 (1.18–1.39)	12.2 (8.2–16.2)
2006	52.1 (48.8–55.5)	40.2 (36.8–43.6)	1.30 (1.17–1.44)	11.9 (7.1–16.7)
2008	52.9 (48.4–57.4)	38.4 (33.9–43.0)	1.38 (1.19–1.59)	14.5 (8.1–20.9)
2009	51.4 (48.3–54.5)	38.5 (35.4–41.6)	1.33 (1.21–1.48)	12.9 (8.5–17.3)
2010	51.7 (48.4–55.0)	37.8 (34.3–41.3)	1.37 (1.22–1.53)	13.9 (9.0–18.7)
2011	52.2 (48.8–55.7)	38.0 (34.5–41.6)	1.37 (1.22–1.54)	14.2 (9.2–19.2)
2012	53.5 (47.6–59.4)	35.5 (32.0–39.1)	1.51 (1.30–1.75)	18.0 (11.1–24.9)

*Source*: *NSW Population Health Survey*, *Centre for Epidemiology and Evidence*, *NSW Ministry of Health*.

^a^ Defined as having two or more individual health risk behaviours.

^b^ Estimates for 2007 are not reported because the survey split questionnaire design used in this year was not consistent with other years.

^c^ Estimates adjusted for age and sex structure of NSW population and probability of selection for inclusion in the survey; estimates standardised to age and sex structure of NSW population for 2012; estimates exclude persons with responses “Don’t know”, “Refused” or “Not asked”.

^d^ Reference group is the least disadvantaged group.

^e^ For education, includes persons with at most a school certificate; for IRSD, includes persons who belonged to the 5^th^ quintile.

^f^ For education, includes persons who completed a university degree or higher; for IRSD, includes persons who belonged to the 1^st^ quintile.

## Discussion

Tackling health inequalities has been a goal of many governments and health organisations worldwide [9]. As lifestyle risk behaviours are important contributing factors to inequalities in health outcomes, it is important to include multiple behavioural risk factors in population health surveillance. Built upon previous surveillance work in NSW, which reported a socioeconomic gap in a state-wide “Dashboard of Indicators” in 2002, this study is among the first to find a widening gap in health risk behaviours by socioeconomic status in Australia. This increasing socioeconomic gap was particularly prominent in smoking, fruit and vegetable intake and the summary lifestyle risk indicator.

From 2002 to 2012, NSW has observed an overall improvement in health behavioural profiles at the population level, indicating net sum effects of prevention efforts, especially for physical activity and smoking. The proportion of adults who engaged in multiple risk factors also declined over time. Steadily declining smoking in NSW mirrors the national trends in Australia [30]. The improving physical activity levels in NSW, however, appear to deviate from the national trends, which conversely show a slight decrease over time [31]. The fluctuating trends in fruit and vegetable intake and at-risk alcohol use in NSW resemble those at the national level [32].

In the past decade, many public health initiatives targeting these risk behaviours have been implemented in NSW and nationally, which may have contributed to the overall improved profiles of lifestyle risk behaviours. For example, the NSW Government has led a comprehensive and sustained approach to tobacco control, incorporating extensive regulation, prevalent social marketing and an accessible and well-advertised cessation programs [33].

Triggers for the improvements in physical inactivity are less clear, with initiatives in NSW tending towards healthy lifestyle social marketing such as “Measure Up” [34] (launched nationally in 2008) or the Get Healthy Service [35] (a telephone-based coaching service launched in NSW in 2009), both of which could not explain earlier improvement in physical activity. However, it seems likely that the ‘cluttered environment’ of physical activity-promoting messages from a myriad of sources may have contributed [36]. Meanwhile, a number of programs, mostly using social marketing and social support approaches, have been implemented to promote healthy eating, such as “Go for 2&5”, which was launched in 2005 to promote fruit and vegetable consumption to parents and carers of children [37]. An evaluation of the campaign showed that fruit and vegetable consumption increased in the 2007 run, but remained steady during 2008 [38]. The approaches to reducing alcohol-related risks have targeted young people, binge-drinking and drink-driving rather than focusing on proven strategies to curb harmful consumption in the wider population [39]. This might explain the lack of steady decrease in higher than recommended alcohol use at the population level.

The improvement in overall lifestyle behaviours, particularly in smoking and physical activity at the population level in NSW is a public health success. Conversely, improvements in lifestyle risk behaviours have occurred at a much faster pace in the least disadvantaged subpopulations compared with the most disadvantaged, resulting in a widening gap over time. Trends by area-level measures of socioeconomic disadvantage showed a similar widening gap between the least and most disadvantaged areas.

Such a “trade-off” between population-level improvement and widening inequalities is a common dilemma in public health. More than 40 years ago, Julian Tudor Hart proposed the “inverse care law”, stating that the availability of good medical care tended to inversely correlate with the need of the population [40]. In 2000, Victora and colleagues postulated a corollary of the “inverse care law” which hypothesised that quality public health programmes were more available and being utilised more by those who needed them the least (the “inverse equity hypothesis”) [41]. This may be the case for new public health interventions introduced at the population level, as proposed by the “diffusion of innovation” theory, wherein new ideas and behaviours were adopted early by those of higher socioeconomic status [42]. Further, the socioeconomic and structural constraints on behaviour change may be greater among disadvantaged people.

### Implications for future public health priorities

At the global level, health inequalities have become a World Health Organization priority, as part of the Health 21 Strategy to reduce the health gap between socioeconomic groups by 2020 [43]. Similarly, specific goals have been set by governments in the United States, England, Scotland, Ireland, and Finland [9]. In Australia, despite an apparent focus on health equity, substantial socioeconomic gaps in health behaviours and outcomes continue over time [4], and further, no explicit quantifiable goals for reducing health inequalities have been proposed by the government.

Geoffrey Rose proposed that a population approach to health promotion is radical and attempts to remove the “underlying causes of disease”, but should be complemented by a high-risk approach to achieve larger risk reductions among smaller populations [44]. Capewell and Graham argued that high-risk “argentic” interventions alone, which require material or psychological resources of individuals, tend to lead to widening inequalities [45]. To promote population-level health improvement without widening socioeconomic gaps, Australia and other countries may consider adopting a dual strategy that integrates sufficient population-wide and high-risk approaches [44, 45].

### Strengths and limitations

This study is among the first in Australia to examine trends in combined lifestyle risk by socioeconomic status. Continuous data collection among population representative samples through the NSW Adult Population Health Survey provides a unique opportunity for this inquiry. Strengths of this study include measuring both individual- and area-level socioeconomic status, both single and summary risk factors, and presenting indicators of both absolute and relative inequalities. Limitations of the study included having only self-reported measures, which nevertheless is a common practice in population health surveillance. The response rate of around 45% may raise concern for selection bias. However, the response and cooperation rates remained stable overtime, and post-stratification weights were applied to account for non-response among certain population subgroups. Therefore, each year’s sample was considered representative of the state’s Local Health Districts [19]. Another potential limitation is using only simple indicators for inequalities instead of sophisticated measures which take into account all SES categories. However, we chose the former approach in the context of a complex survey design, multiple lifestyle risk indicators and repeated measures, as a simple “range” approach provides estimates that are straightforward, interpretable, and can be easily summarised and communicated in a policy setting [8]. Furthermore, the current analysis could be improved by using more comprehensive risk measures, such as incorporating binge-drinking measures in the quantification of alcohol risk, and additional food items in the quantification of dietary risk. Finally, due to competing factors during the study period, it is difficult to single out effects of specific public health initiatives. As well, due to the lack of international comparative studies, it is difficult to determine whether local policies have been more or less effective in reducing inequalities in health risk [15]. Thus, the current study is limited to a description of health inequalities without testing specific mechanisms though which inequalities worsen.

### Conclusions

Based on population representative samples from NSW, Australia, this study found that between 2002 and 2012 the overall profile of lifestyle risk behaviours has improved, particularly in smoking and physical activity. However, the socioeconomic gaps in the overall lifestyle risk indicator and in smoking and dietary behaviour have widened in terms of both absolute and relative inequalities. Australian governments should set explicit quantifiable goals for reducing health inequalities, monitor population-level indicators, and adopt population-wide strategies to improve health behaviours and outcomes, particularly among those who are most in need.
